# Efficacy of Physical Exercise in Ameliorating Circadian Rhythm Disruption-Related Intestinal Inflammation: A Benefit Correlated with Reduced Chenodeoxycholic Acid

**DOI:** 10.3390/cells15171537

**Published:** 2026-08-26

**Authors:** Yimin Du, Erxing Wang, Yueqi Leng, Qianqian Wang, Jiacen Sun

**Affiliations:** 1 College of Food Science and Technology, Shanghai Ocean University, Shanghai 201306, China; m240401090@st.shou.edu.cn (Y.D.); wang_erxing@163.com (E.W.); 2 Naval Medical Center, Naval Medical University (Second Military Medical University), Shanghai 200433, China; iamlyq0112@smmu.edu.cn

**Keywords:** physical exercise, intestinal inflammation, circadian rhythm disruption, chenodeoxycholic acid

## Abstract

**Highlights:**

**What are the main findings?**

- Physical exercise could alleviate circadian rhythm disruption-related intestinal inflammation, and this has a correlation with reduced biosynthesis of chenodeoxycholic acid.
- The beneficial effect of physical exercise in ameliorating circadian rhythm disruption-related intestinal inflammation may be associated with upregulated intestinal clock gene expression, while the genera *Prevotella* and *Lactococcus* in gut microbiota were potential mediators of this.

**What are the implications of the main findings?**

- An anti-inflammatory effect of physical exercise with preliminarily demonstrated relevance to reduced chenodeoxycholic acid was suggested by an animal experiment and a combination of transcriptome, microbiome, and metabolome analyses.
- Adequate physical activity may play an important role in preventing gastrointestinal disorders mediated by circadian rhythm disruption caused by the modern lifestyle.

**Abstract:**

Circadian rhythm disruption (CRD)-related intestinal inflammation is a significant cause of gastrointestinal disorders. Although the benefits of physical exercise have been investigated, its preventive and therapeutic roles in CRD-related intestinal inflammation remain ambiguous. Given the critical role of gut microbiota in regulating intestinal homeostasis and the inflammatory response to external stimuli, we hypothesized that gut microbiota could mediate the anti-inflammatory efficacy of physical exercise on intestinal inflammation. Initially, a two-sample bidirectional Mendelian randomization (MR) was conducted on the causality between physical exercise, intestinal inflammation, and gut microbiota. Subsequently, the preventive efficacy was evaluated in a CRD mouse model. Next, a potential anti-inflammatory mechanism was explored through transcriptomic sequencing, 16S rRNA sequencing, and fecal metabolome analysis. The results of the MR suggested alleviated intestinal inflammation from gut microbiota modulated by physical exercise. Ameliorated intestinal injury and inflammation were observed in exercising CRD mice, which was dampened by oral chenodeoxycholic acid (CDCA) administration. Then, comprehensive analysis indicated that the benefit of physical exercise may be associated with enhanced intestinal clock gene expression and the genus *Prevotella*. Our findings indicate the potential for the effects of physical exercise to be relevant to reduced CDCA; they also emphasize the benefits of undertaking adequate physical activity in maintaining a healthy condition within a modern lifestyle that involves various risk factors.

## 1. Introduction

Circadian rhythm disruption (CRD) is a prevalent characteristic of the modern lifestyle and is associated with various gastrointestinal disorders, including inflammatory bowel diseases and irritable bowel syndrome [1,2,3]. Due to the crucial role of the circadian system in the regulation of metabolism and the immune system, a significant consequence of CRD in the gastrointestinal system is prolonged intestinal inflammation that disrupts immunoregulation, barrier integrity, and epithelia proliferation [4,5,6,7]. Commensal gut microbiota is a critical component of the gastrointestinal system and is intertwined with the circadian system within its host [8,9]. A disrupted circadian rhythm could alter gut microbiota and lead to various negative impacts, such as a decreased abundance of anti-inflammatory bacteria, increased microbial endotoxin production, and perturbed biosynthesis of beneficial microbiome metabolites and microbiome–host communication [9]. Moreover, intestinal inflammation could be promoted by dysbiosis, which renders the host more susceptible to pathogen colonization due to a reduced diversity and proportion of probiotics [10].

Gastrointestinal disorders are highly prevalent. More than 40% of people have been affected at any one point in time, and nearly two-thirds of patients have chronic or fluctuating symptoms [11]. Current therapeutics in gastrointestinal disorders are somewhat limited by various shortages. During pharmaceutical therapy, drug dependency, diminished efficiency, and potential side effects in long-term administration have been reported by previous studies [12,13]. Improved therapies such as biological agents and fecal microbiota transplantation are costly and limited by individual variations or long-term safety [14,15]. Therefore, novel management approaches are still required, especially personalized and targeted ones addressing underlying pathophysiology and psychology [11].

Physical exercise has been considered a beneficial activity for health since ancient times, and its therapeutic effects against various diseases, such as hypertension, coronary heart disease, and diabetes, have been reported in previous investigations [16,17,18]. The association between the improved diversity of gut microbiota and several kinds of physical activities has been revealed by numerous investigations on physical exercise [19]. During regular physical exercise, cross-talk between active skeletal muscles and the gut microbiota could boost immune function, improve nutrient absorption, and facilitate metabolic regulation in the host [20]. Furthermore, the importance of gut microbiota in mediating the beneficial efficacy of physical exercise has been suggested by several studies in recent years, aiding conditions such as osteopenia and myocardial infarction [21,22]. The efficacy of physical exercise in alleviating the symptoms of gastrointestinal disorders has been studied by various clinical studies, with effects including improved physical fitness, reduced pro-inflammatory cytokines, and ameliorated mental health conditions [23,24,25]. However, its therapeutic effect on intestinal inflammation caused by CRD still remains ambiguous, either in terms of the underlying mechanism or its influence on gut microbiota altered by a perturbed circadian system.

To this end, the main purpose of this study is to explore the efficacy of physical exercise in ameliorating CRD-related intestinal inflammation and its potential mechanism. Considering the role of gut microbiota in maintaining intestinal homeostasis and response to external stimuli that are described hereinabove, we hypothesized that gut microbiota could mediate the anti-inflammatory efficacy of physical exercise in the treatment of gastrointestinal disorders. First, the causality of physical activity, intestinal inflammation, and gut microbiota was analyzed by a bidirectional two-sample Mendelian randomization (MR) approach. Then, the efficacy of physical exercise on anti-inflammation in the gastrointestinal (GI) tract was evaluated using a CRD mouse model. Next, the potential mechanism for the efficacy of physical exercise was explored by a multi-omics integration analysis among the intestinal transcriptome, gut microbiota, and fecal metabolome. Our findings indicate a feasible efficacy of physical activity in ameliorating CRD-related intestinal inflammation, which is associated with reduced biosynthesis of CDCA. Enhanced intestinal clock gene expression and physical exercise-induced alterations in gut microbiota may also contribute to the beneficial effects of physical activity. The importance of adopting an appropriate physical exercise strategy is also supported by its beneficial effects in alleviating gastrointestinal health problems caused by circadian rhythm disruption, which primarily arise from the modern lifestyle.

## 2. Materials and Methods

### 2.1. Mediation Mendelian Randomization Study

Eight datasets from the IEU OpenGWAS, comprising a total of 2,596,825 samples, were used as exposure factors (Table A1). A dataset (GWAS ID: GCST90014444) from the NHGRI-EBI GWAS Catalog was used as the outcome factor. A total of 211 gut microbiota datasets, including 9 phyla, 16 classes, 20 orders, 35 families, and 131 genera, were obtained from the latest GWAS summary data of the MiBioGen consortium [26]. The Mediation Mendelian randomization study was conducted with reference to previous studies that have conducted Mendelian randomization and mediation analysis [27,28,29]. The analysis of MR was performed with R (version 4.4.2) using the package ggplot2 (version 3.5.1) and the TwoSampleMR (version 0.6.17). SNPs not in linkage disequilibrium (R2 < 0.001, kb < 1000 kb) and that are significantly associated with exposure (*p* < 1 × 10^−5^) were selected as instrumental variables (IVs). IVs with an *F*-value < 10 were excluded to minimize weak instrument bias.

The causality between exposure factors and outcome factors was primarily estimated by the inverse variance weighted (IVW) approach, supplemented by results from 4 other complementary approaches (MR Egger, Weighted median, Simple mode, and Weighted mode). Scatter plots were generated using the ggplot2 package based on the estimation results from the causality analysis. Additional complementary methods were employed to evaluate the stability of the causality analysis. The contribution of individual SNPs to the outcome was estimated using the TwoSampleMR package function “mr_forest_plot”, while the total effects were estimated using the IVW approach. Direction of the causal relationship (Steiger *p*-Val) was checked using the TwoSampleMR package function “steiger_filtering”. In sensitivity estimation, heterogeneity and pleiotropy were estimated using the TwoSampleMR package functions “mr_heterogeneity” and “mr_pleiotropy_test”, respectively. The heterogeneity (Q *p*-Value) was primarily estimated using the IVW approach, supplemented by the results from the MR Egger approach. The pleiotropy (P *p*-Value) was estimated by the MR Egger approach. Randomness was estimated by a combination of SNP effects and the standard error using the funnel plot. Robustness of the causal relationship were assessed using the TwoSampleMR package function “mr_leaveoneout”. The causality between physical activity and intestinal inflammation, gut microbiota and intestinal inflammation, and physical activity and gut microbiota were estimated using the above-described analysis strategies. Exposures with no significant heterogeneity and no significant horizontal pleiotropic effects were encompassed in the mediation analysis.

Gut microbiota and physical activity associated with intestinal inflammation were included in the Two-step MR for Mediation Analysis. Initially, the overall effect (β-all) of physical activity on intestinal inflammation was estimated, followed by the effect of physical activity on gut microbiota (β-1) and the effect of gut microbiota on intestinal inflammation (β-2). The mediation effect (β-ind) was calculated as follows: β-ind = β-1 × β-2, while the direct effect (β-dir) = β-all − β-ind. A mediation test was also performed to obtain a significance level of the mediation effect.

### 2.2. Animal Models

Initially, the 40 C57BL/6 mice (male, 8 weeks old) included in this study were housed in an animal facility under standard conditions (22 ± 2 °C, 55 ± 5% humidity) for 1 week to acclimate to the environment. The mice were then randomly divided into four groups, with 10 mice per group. The number of mice in each group was selected with reference to numerous previous studies on physical exercise; a group size of 10–12 mice was considered ideal for a balance of statistical power and the rule of 3Rs. These groups included the following: Group LD-Sed: normal circadian mice without exercise training served as the control group; Group LD-Ex: normal circadian mice with low-intensity exercise training; Group CD-Sed: CRD mice without exercise training; Group CD-Ex: CRD mice with low-intensity exercise training. LD mice were housed under a standard 12 h light/dark cycle (8:00 a.m.–8:00 p.m.). CRD mice were housed in specialized cupboards for 5 weeks to induce intestinal inflammation. The light-cycle for CRD mice was shifted by 8 h every 3 days, as previously described [30]. Exercise training was performed with reference to a previous description [31]. Briefly, mice were trained on a treadmill for 60 min per day from the beginning to the end of the experiment. The speed was initially set at 6 m/min and increased by 1 m/min per day until reaching 12 m/min. Food and water were available ad libitum to all mice throughout the experiment. In the causal study on the effect of CDCA, 8 mice were included and housed under the same conditions as the CD-Ex group. Furthermore, 4 mice were assigned to the control group (Saline) and administered a gavage of saline and cosolvent, while the other 4 mice were administered a gavage of saline, cosolvent, and CDCA (2.5 mg/mL) as the experimental group (CDCA). All gavages were performed once per day (0.8 mL/100 g) until the end of the experiment.

Mice were euthanized by intraperitoneal injection of 20% ethyl carbamate (0.9 mL/100 g) before sample collection. All experimental protocols adhered to ethical guidelines and were approved by the Institutional Animal Care and Use Committee (IACUC) of the Naval Medical University (Protocol No. NMU-20210901).

### 2.3. Histopathological Analysis

The mice used for histopathological analysis were perfused through the heart with 0.9% sodium chloride solution (40 mL per mouse), followed by phosphate-buffered solution (PBS) with 4% paraformaldehyde (20 mL per mouse). Next, excised intestinal tissues were fixed in 4% paraformaldehyde solution at 4 °C overnight. The fixed intestinal tissues were embedded in paraffin, cut into sections with a thickness of 4 μm, and stained with hematoxylin and eosin (H&E), or with iron hematoxylin, ponceau, and aniline blue (Masson’s trichrome).

### 2.4. Real-Time qPCR

Excised intestinal tissues were washed with PBS and snap-frozen in liquid nitrogen overnight. Extraction and reverse transcription of total RNA were performed using the RNA Extraction Reagent Kit (Sangon Biotech, Shanghai, China) and the PrimeScript RT Master Mix (Sangon Biotech, Shanghai, China) following the manufacturer’s instructions, respectively. The qPCR was then performed on a QuantStudio 6 Flex system (Thermo Scientific, Waltham, MA, USA) using TB Green Premix Ex Taq II (Sangon Biotech, Shanghai, China). Cycling conditions were as follows: 95 °C for 30 s, followed by 40 cycles of 95 °C for 5 s and 60 °C for 30 s. The primers used in this study are listed in Appendix A, and Gapdh was selected as the reference gene (Table A2).

### 2.5. Western Blotting

Intestinal tissues were lysed using RIPA lysis buffer (Beyotime, Shanghai, China) in an ice-water bath. Protein concentrations were then quantified using the BCA protein assay reagent kit (Beyotime, Shanghai, China). The collected proteins were heated at 100 °C for 10 min in a metal dry bath and diluted to a uniform concentration (3 μg/μL) with 5 × SDS-PAGE protein loading buffer (Beyotime, Shanghai, China). Proteins were separated by gel electrophoresis and transferred onto PVDF membranes (0.45 μm; Merck Millipore, Darmstadt, Hessen, Germany). The membranes were blocked with 5% skim milk for 2 h at room temperature, followed by incubation with primary antibodies at 4 °C overnight. Subsequently, the membranes were incubated with corresponding secondary antibodies for 2 h at room temperature, and the bands were visualized using the Omni-ECL™ Femto Light Chemiluminescence Kit (Epizyme Biotech, Shanghai, China) on a Tanon 5200 imaging system (Tanon, Shanghai, China). The antibodies used in Western blotting are listed in Appendix A, and Gapdh was chosen as the internal control (Table A3).

### 2.6. Immunofluorescence Stain

Intestinal tissues were collected and fixed using the same approach as described for histopathological analysis. The fixed intestinal tissues were stored at −20 °C overnight and then sectioned at a thickness of 10 μm. The sections were blocked with 5% bovine serum albumin (BSA) for 2 h at room temperature, followed by incubation with primary antibodies at 4 °C overnight. Subsequently, the sections were incubated with corresponding fluorescent conjugated secondary antibodies for 2 h at room temperature and imaged using a fluorescence microscope (Leica, Wetzlar, Hessen, Germany). The antibodies used in immunofluorescence staining are listed in Appendix A (Table A3).

### 2.7. Transcriptomic Sequencing and Analysis

Collection of the intestinal tissues and total RNA extraction were performed using the same approach as in real-time qPCR. Library construction and sequencing of the transcriptome were conducted by OE Biotech Co., Ltd. (Shanghai, China). Purity and quantification were evaluated using the NanoDrop 2000 spectrophotometer (Thermo Scientific, Waltham, MA, USA). Then, RNA integrity was assessed using the Agilent 2100 Bioanalyzer (Agilent Technologies, Santa Clara, CA, USA). Next, the transcriptome libraries were constructed using the TruSeq Stranded mRNA LT Sample Prep Kit (Illumina, San Diego, CA, USA).

The libraries were sequenced on an Illumina HiSeq X Ten platform. Furthermore, 150 bp paired-end reads and approximately 48.53 M raw reads for each sample were generated. Raw reads were processed by Trimmomatic (version 0.36) to remove low-quality reads [32]. About 47.66 M clean reads for each sample were retained for bioinformatic analysis. The clean reads were mapped to the mouse genome (GRCm39), and the FPKM of each gene was calculated. Differential expression analysis was performed using the R package DESeq (1.8.3), while a *p*-value < 0.05 and fold change > 2 or fold change < 0.5 were set as thresholds. GO enrichment and KEGG pathway enrichment of DEGs were performed using R based on the hypergeometric distribution.

### 2.8. 16S rRNA Sequencing and Analysis

In 16S rRNA sequencing, fecal samples were collected from the cecum, then snap-frozen, and stored at −80 °C before extraction and isolation. 16S rRNA sequencing was conducted by OE Biotech Co., Ltd. (Shanghai, China). The concentration and integrity of the extracted DNA were measured using a NanoDrop 2000 spectrophotometer (Thermo Fisher Scientific, Waltham, MA, USA) and agarose gel electrophoresis, respectively. The V3-V4 hypervariable regions of the bacterial 16S rRNA gene in extracted bacterial DNA were amplified using universal primer pairs (343F: 5′-TACGGRAGGCAGCAG-3′; 798R: 5′-AGGGTATCTAATCCT-3′). The reverse primer contained a sample barcode, and both primers were connected with an Illumina sequencing adapter. The amplicons were purified with Agencourt AMPure XP beads (Beckman Coulter Co., Indianapolis, IN, USA) and quantified using the Qubit dsDNA assay kit. The concentrations of DNA in samples were adjusted to equal amounts and pooled for subsequent sequencing.

Sequencing was conducted on an Illumina NovaSeq6000 with two paired-end read cycles of 250 bases each. (Illumina Inc., San Diego, CA, USA; OE Biotech Company; Shanghai, China). Ambiguous bases and low-quality sequences were cut off using Trimmomatic (version 0.36) before assembly [32]. Preprocessed paired-end reads were assembled and processed using FLASH (version 1.2.11), QIIME (version 1.8.0), and VSEARCH (version 1.9.0). Then, operational taxonomic units (OTUs) were generated from clean reads [33,34,35]. The representative read of each OTU was selected using QIIME, then annotated and blasted using the RDP classifier (confidence threshold: 70%) with the SILVA database (version 132) [36]. Microbial diversity was estimated using alpha diversity, including Chao1 and the Shannon and Simpson indices, as well as beta diversity, including principal coordinates analysis (PCoA) and the linear discriminant analysis effect size (LEFSe).

### 2.9. Untargeted Metabolomic Analysis

Intestinal contents were collected from the excised intestinal tissues of euthanized mice. For each sample, 30 mg of intestinal contents was mixed with L-2-chlorophenylalanine and 25% methanol, then extracted sequentially by grinding and ultrasonication, and purified by centrifugation. The extracts were stored at −80 °C until further analysis.

Metabolic profiling of extracted samples was performed using liquid chromatography–mass spectrometry (LC-MS) on a Dionex Ultimate 3000 RS UHPLC coupled with a Q-Exactive plus quadrupole orbitrap mass spectrometer equipped with a heated electrospray ionization (ESI) source (Thermo Fisher Scientific, Waltham, MA, USA). Chromatographic separation was carried out on the column ACQUITY UPLC HSS T3 (100 mm × 2.1 mm, 1.8 um; Waters, Milford, MA, USA) with mobile phases consisting of 0.1% formic acid in water, acetonitrile at 45 °C, and a flow rate of 0.35 mL/min. QCs were injected at regular intervals to assess repeatability. Raw reads were processed using software Progenesis QI V2.3 (Nonlinear, Dynamics, Newcastle, UK), and further analyses were performed using R.

### 2.10. Statistical Analysis

The results of immunofluorescence staining and Western blotting were quantified using Fiji (version 2.16.0) [37]. All statistical analyses excluded from the MR study and the bioinformatic analysis were performed with GraphPad Prism V.9.0.0 (GraphPad Software, San Diego, CA, USA). The results of the histopathological analysis, immunofluorescence (IF) staining, real-time qPCR, and Western blotting were presented as individual points (the black dots in histograms) + mean ± standard deviation (SD). Statistical significance of the differences was estimated using an unpaired Student’s *t*-test (comparison between the saline and CDCA groups) or two-way ANOVA (comparison among the LD-Sed, LD-Ex, CD-Sed, and CD-Ex groups) followed by Tukey’s post hoc test. A *p* < 0.05 was considered statistically significant, while * *p* < 0.05; ** *p* < 0.01; *** *p* < 0.001, and **** *p* < 0.0001.

## 3. Results

### 3.1. MR and Mediation Analyses for the Mediation Role of Gut Microbiota in the Causal Relationship Between Physical Exercise and Intestinal Inflammation

**Figure 1:**
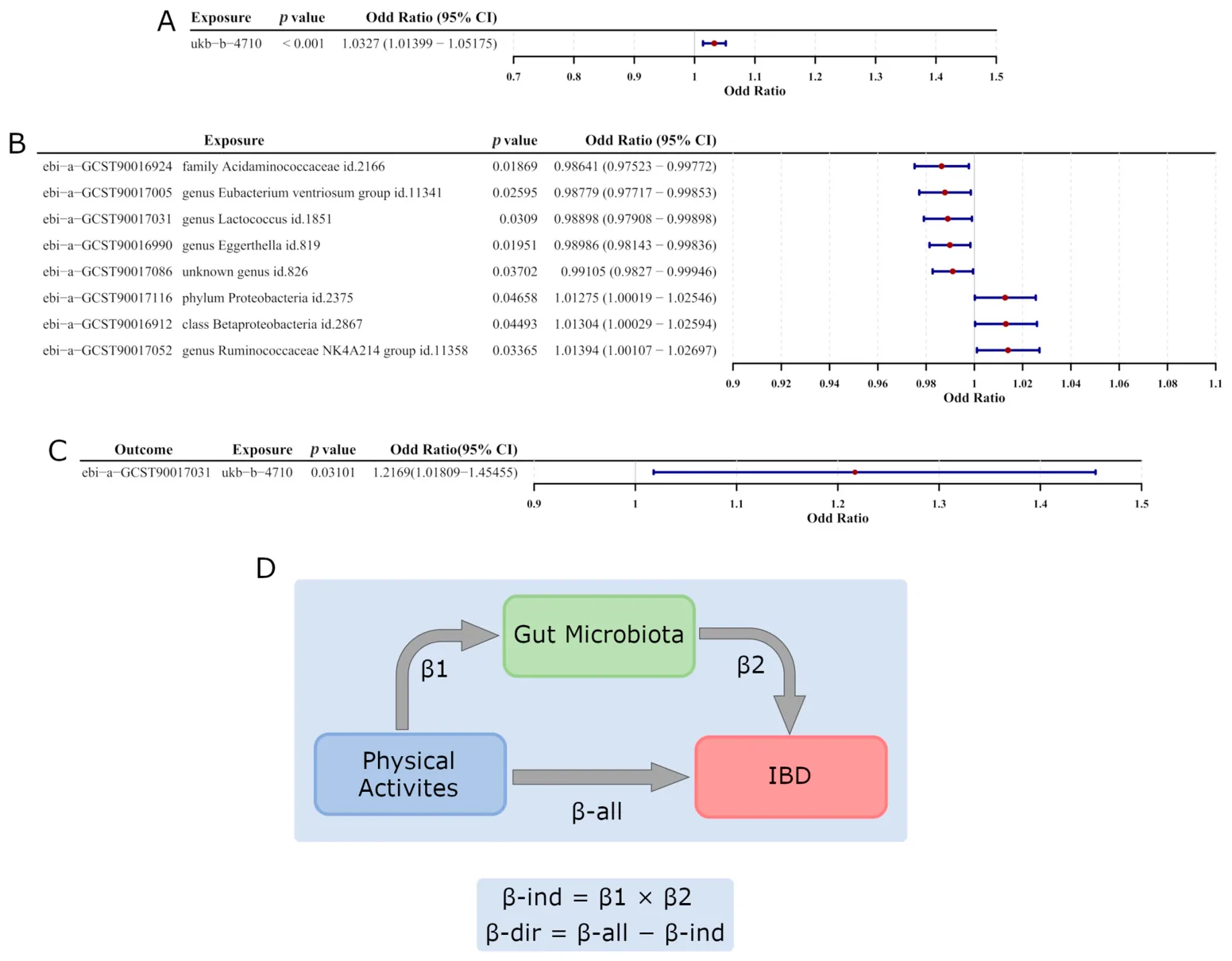
The mediation analysis for gut microbiota in the pathway from physical activity to intestinal inflammation. MR results for the datasets of physical activity (**A**) and gut microbiota (**B**) involved in the mediation analysis. (**C**) Results of the MR analysis of causality between physical activity (number of days/week of moderate physical activity 10+ min || id:ukb-b-4710) and gut microbiota (genus Lactococcus id.1851|| id:ebi-a-GCST90017031). (**D**) Study design of the mediation analysis. The red dots and blue lines represent odds ratios (OR) and corresponding 95% confidence intervals (CI) for the outcomes. β-all: overall effect; β-ind: mediation effect from gut microbiota; β-dir: direct effect of physical activity.

In the initial analysis, potential causality between eight datasets of physical activity and a dataset (inflammatory bowel disease || id:GCST90014444) of disease relevant to CRD-related intestinal inflammation were examined. A total of 954 SNPs from exposures with a sufficient level of effect size were used as IVs (Table S1). Causal effects were identified between five physical activity datasets and intestinal inflammation: Time spent watching television (ukb-b-5192, OR = 1.034 [1.0198, 1.049], *p* < 0.001), number of days/week of moderate physical activity 10+ min (ukb-b-4710, OR = 1.033 [1.014, 1.05], *p* < 0.001), and moderate to vigorous physical activity levels (ebi-a-GCST006097, OR = 1.0633 [1.019, 1.1095], *p* = 0.00465) were risk factors for intestinal inflammation. Walking for pleasure (ukb-b-7337, OR = 0.943 [0.904, 0.984], *p* = 0.0067) and vigorous physical activity (ebi-a-GCST006098, OR = 0.96 [0.927, 0.995], *p* = 0.025) were protective factors for intestinal inflammation (Table 1). A stable correlation between physical activity and intestinal inflammation was supported by scatterplots (Figures S1–S8). The sensitivity of the causality analysis between physical activity and intestinal inflammation was assessed using the pleiotropy and heterogeneity tests. No significant horizontal pleiotropic effect was found for ukb-b-7337 (P *p*-value= 0.720), ebi-a-GCST006098 (P *p*-value = 0.320), or ukb-b-4710 (P *p*-value = 0.196) (Table 1). Only ebi-a-GCST006097 (Q *p*-value = 0.363) and ukb-b-4710 (Q *p*-value = 0.729) were datasets associated with intestinal inflammation without significant heterogeneity (Table 1). The dataset ukb-b-4710 was the only physical activity dataset considered optimal for the mediation analysis (Figure 1A). Its causal effect was further corroborated by funnel plots, analysis of MR effect sizes of SNPs, and leave-one-out sensitivity analysis (Figures S9–S11).

A total of eight gut microbiota datasets were associated with intestinal inflammation, among which five datasets were associated with reduced inflammation levels and three datasets were associated with increased inflammation levels (Table 2 and Table S2, Figure 1B and Figures S12–S19). MR analysis was conducted between physical activity (number of days/week of moderate physical activity 10+ min || id:ukb-b-4710) and gut microbiota datasets associated with intestinal inflammation. Intensity of physical activity was positively associated with an abundance of genus *Lactococcus* (ebi-a-GCST90017031) (Table 3 and Table S3, Figure 1C and Figure S20). The stability of the causality between the genus *Lactococcus* and intestinal inflammation, as well as physical activity, was further corroborated by the same tests applied to the physical activity–intestinal inflammation causality analysis (Figures S21–S26). Subsequently, a two-step MR analysis was performed for mediation analysis (Figure 1D). Moderate physical activity on intestinal inflammation indicated a greater risk (β-dir = 0.0536) than the overall effect (β-all = 0.0322). Nevertheless, the risk of intestinal inflammation was reduced by 67.38% through the genus *Lactococcus* (β-ind = −0.0216), which was promoted by physical activity (β-1 = 0.196; β-2 = −0.111). The significance of the mediation effect of the genus *Lactococcus* was further assessed using a mediation test (*p* = 0.0444).

### 3.2. Intestinal Inflammation in CRD Mice Could Be Alleviated with Physical Exercise

**Figure 2:**
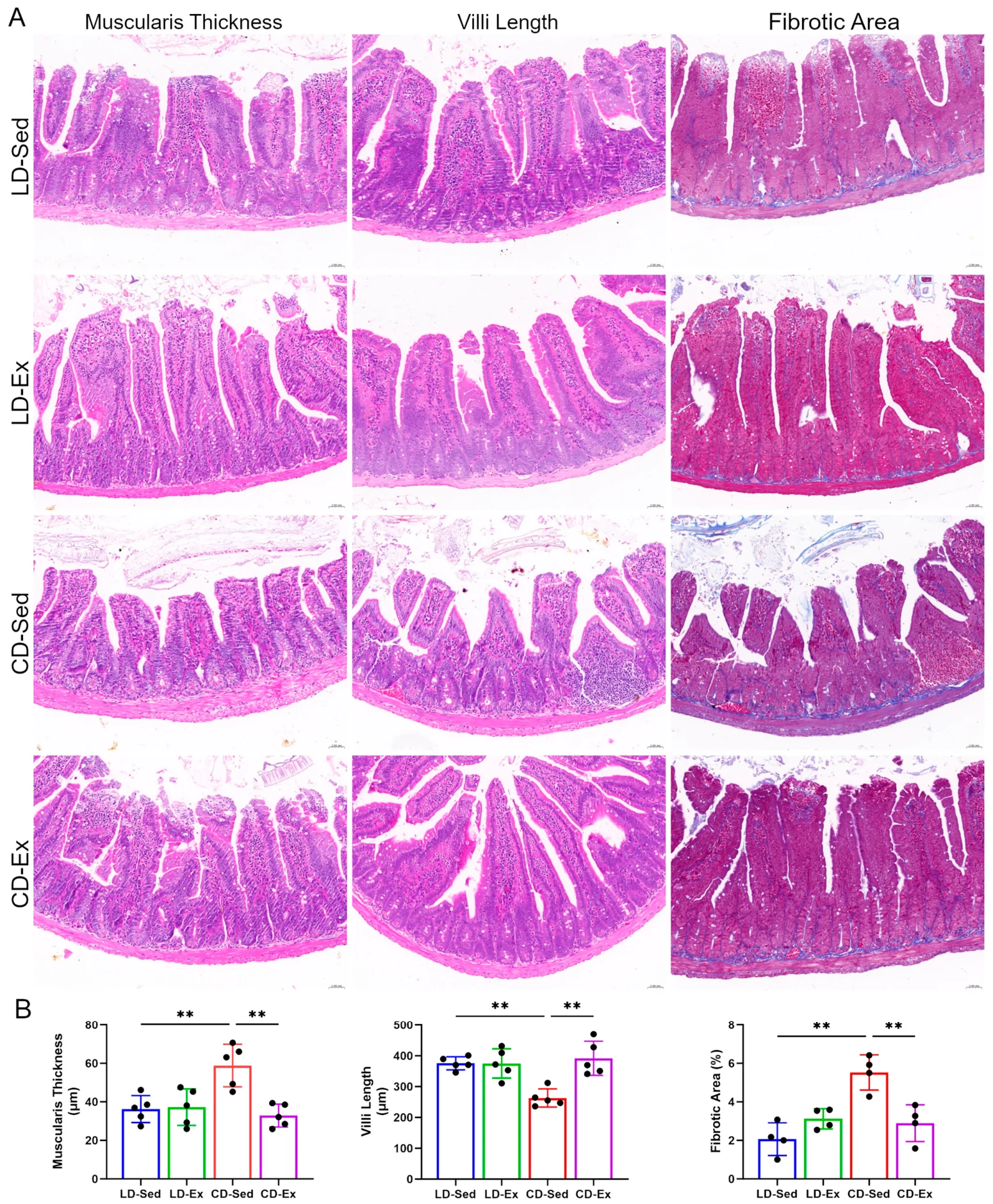
Histopathological analysis of the LD-Sed, LD-Ex, CD-Sed, and CD-Ex groups. (**A**) H&E (**Left**, **middle**) and Masson staining (**Right**) images (20×) of intestinal tissue; quantitative analyses of muscularis thickness (*n* = 5), villi length (*n* = 5) and fibrotic area (*n* = 4) are shown in (**B**). Scale bar = 50 μm. ** *p* < 0.01.

**Figure 3:**
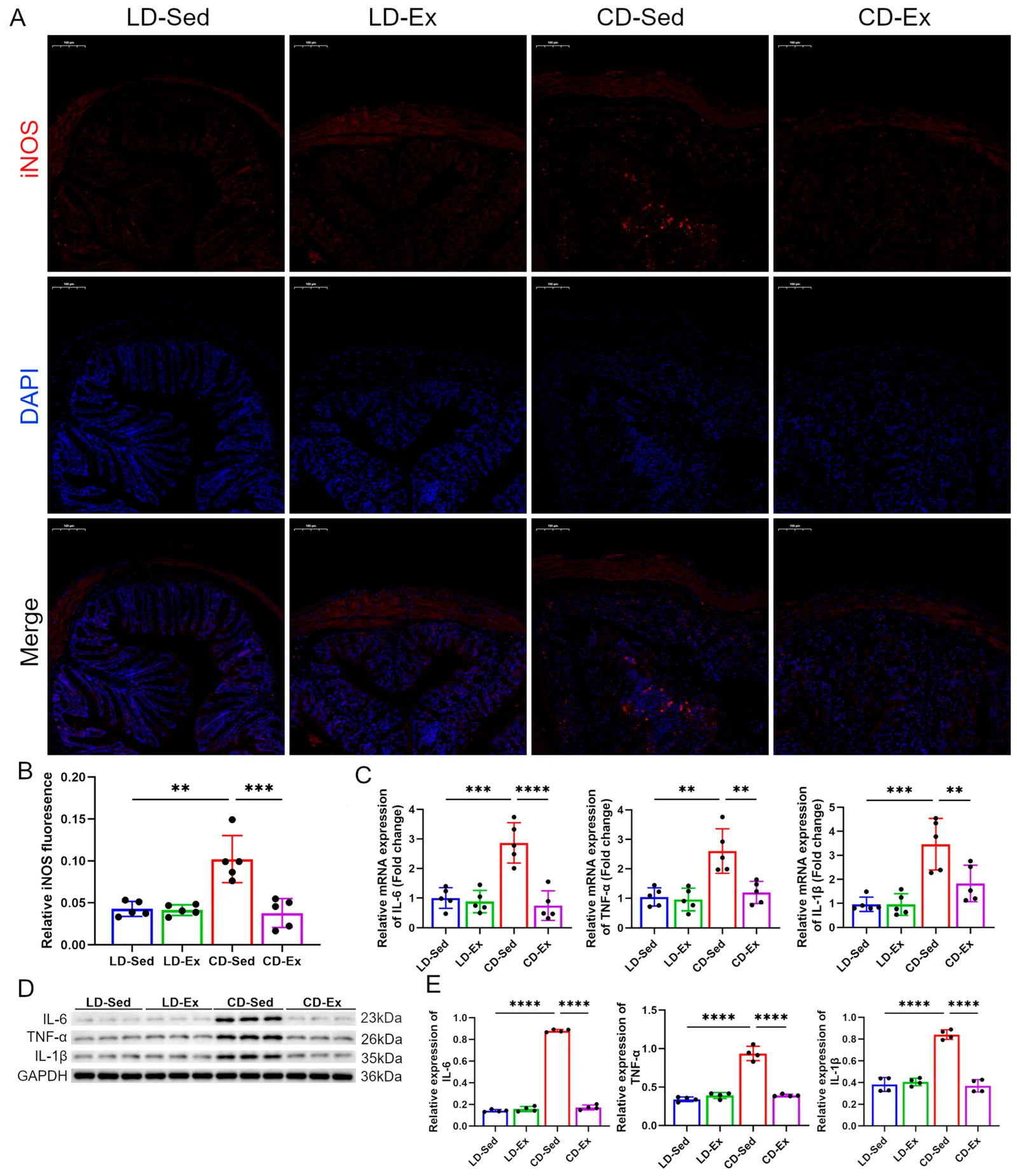
Analysis of inflammatory factors in the LD-Sed, LD-Ex, CD-Sed, and CD-Ex groups. (**A**) Immunofluorescence staining images (20×) of intestinal tissue slices; quantitative results of relative iNOS fluorescence (*n* = 5) are shown in (**B**). (**C**) Relative mRNA expression levels of IL-6, TNF-α, and IL-1β (*n* = 5). (**D**) Western blotting of IL-6, TNF-α, and IL-1β (*n* = 4); corresponding quantitative analysis results are shown in (**E**). Scale bar = 100 μm. ** *p* < 0.01; *** *p* < 0.001, and **** *p* < 0.0001.

**Figure 4:**
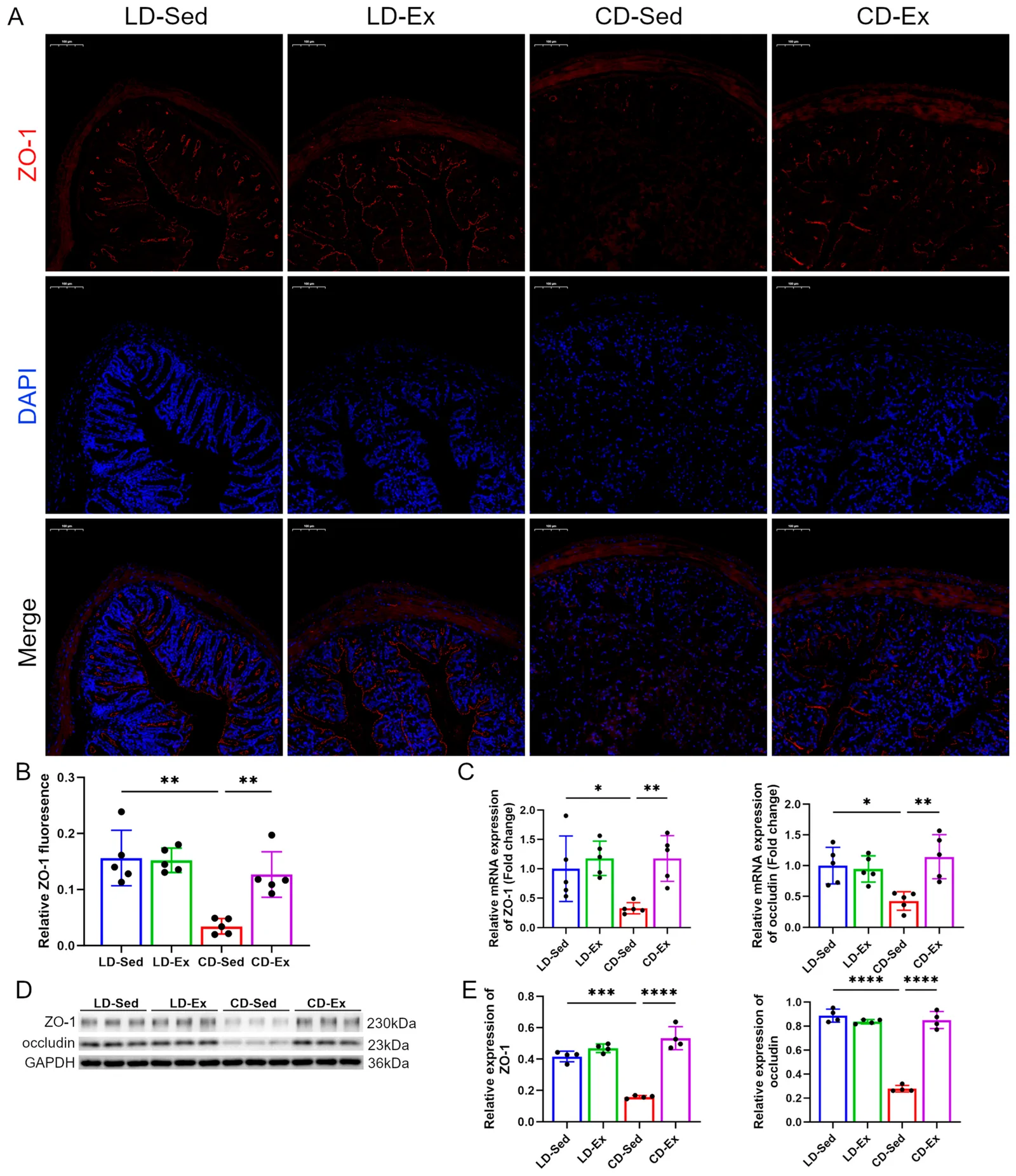
Analysis of intestinal barrier integrity in the LD-Sed, LD-Ex, CD-Sed, and CD-Ex groups. (**A**) Immunofluorescence staining images (20×) of intestinal tissue slices; quantitative results of relative ZO-1 fluorescence (*n* = 5) are shown in (**B**). (**C**) Relative mRNA expression levels of ZO-1 and occludin (*n* = 5). (**D**) Western blot analyses of ZO-1 and occludin (*n* = 4); corresponding quantitative results are shown in (**E**). Scale bar = 100 μm. * *p* < 0.05; ** *p* < 0.01; *** *p* < 0.001, and **** *p* < 0.0001.

To estimate the anti-inflammatory efficacy of physical exercise in the GI tract, a CRD mouse model with exercise training was employed. Histopathological analysis, IF staining, real-time qPCR, and Western blotting were performed in the LD-Sed, LD-Ex, CD-Sed, and CD-Ex groups. Histopathological examination revealed that, compared with the LD-Sed, LD-Ex, and CD-Ex groups, CD-Sed mice exhibited a thicker muscularis, shorter villi, and an expanded fibrotic area (Figure 2A,B). In the assessment of inflammatory factors, CD-Sed mice displayed a higher iNOS fluorescence intensity than the other three groups (Figure 3A,B). The mRNA and protein levels of IL-1β, IL-6, and TNF-α were also increased in CD-Sed mice relative to the LD-Sed, LD-Ex, and CD-Ex groups (Figure 3C–E and Figure S27). In the analysis of intestinal barrier integrity, ZO-1 fluorescence was diminished in the CD-Sed group compared with the LD-Sed, LD-Ex, and CD-Ex groups (Figure 4A,B). Furthermore, the mRNA and protein levels of ZO-1 and occludin were also correspondingly decreased in CD-Sed mice (Figure 4C–E and Figure S28).

### 3.3. Clock Gene Expression in the Intestinal Tract Was Upregulated in Exercised CRD Mice

**Figure 5:**
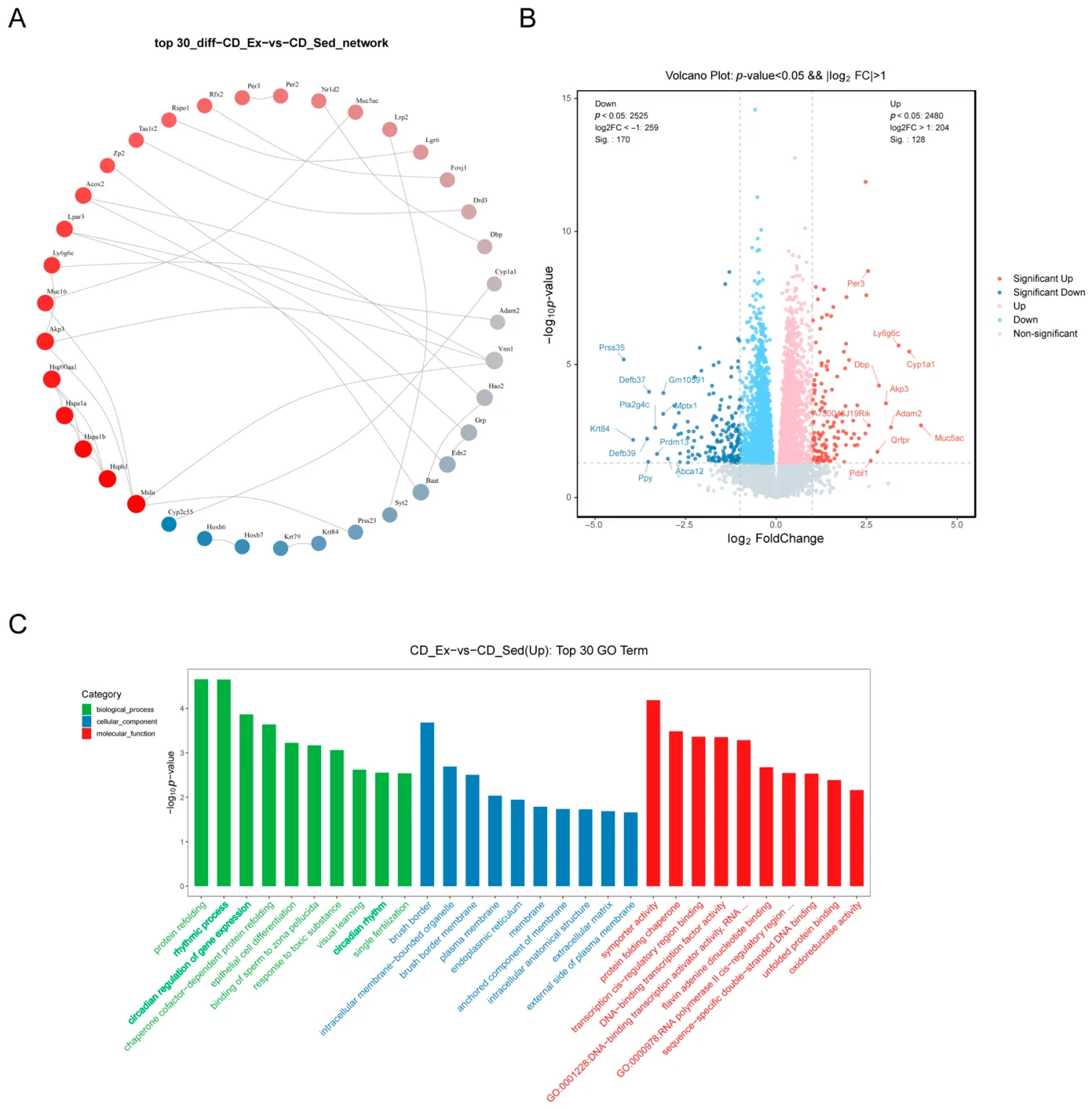
Transcriptomic analysis of the intestinal tract between the CD-Sed and CD-Ex groups. (**A**) PPI network of differentially expressed proteins between the CD-Sed and CD-Ex groups. Node color represents the expression levels of the corresponding proteins, with expression levels increasing from blue to red. (**B**) Volcano plot of DEGs between the CD-Sed and CD-Ex groups; DEGs are defined based on log_2_ fold change >1 or <−1, and −log_10_ *p*-value > 1.301. (**C**) GO enrichment analysis of the top 30 upregulated GO terms; the GO terms valuable to our findings are bolded. *n* = 10 for each group.

To investigate the potential pathways underlying the anti-inflammatory effects of physical exercise, the transcriptome of intestinal tract tissue from mice in the CD-Sed and CD-Ex groups was analyzed. A protein–protein interaction (PPI) network was constructed based on proteins encoded by differentially expressed genes (DEGs) between the CD-Ex and CD-Sed groups. This analysis revealed that the key circadian genes Dbp and Per3 were upregulated, together with their interacting partners Nr1d2 and Per2 (Figure 5A). A total of 298 significant DEGs were identified, of which 128 were upregulated, and 170 were downregulated (Figure 5B, Table S4). The fold changes in Dbp, Per3, Per2, and Nr1d2 in CD-Ex compared with CD-Ex were 7.16, 5.81, 5.55 and 2.67, respectively. Furthermore, Gene Ontology (GO) enrichment analysis of the upregulated GO terms between CD-Ex and CD-Sed revealed upregulation of circadian-related biological processes, including rhythmic process (GO:0048511), circadian regulation of gene expression (GO:0032922), and circadian rhythm (GO:0007623) (Figure 5C).

### 3.4. Abundance of the Genus Prevotella Was Increased in the Gut Microbiota of Exercised CRD Mice

**Figure 6:**
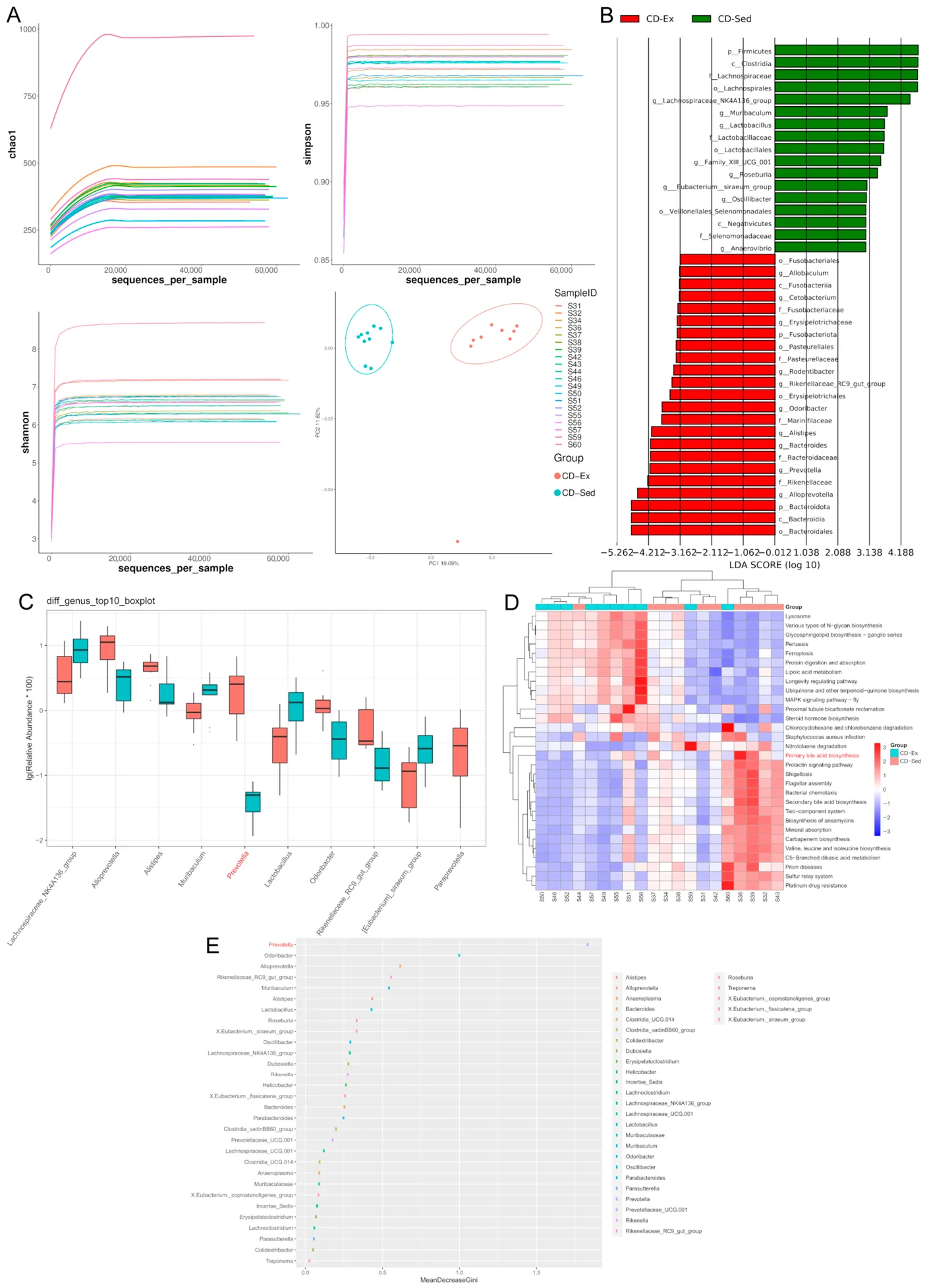
16S rRNA sequencing analysis of the gut microbiota of the CD-Sed and CD-Ex groups. (**A**) Alpha diversity, including the Chao1, Simpson, and Shannon indices, and Beta diversity assessed by PCoA of the CD-Sed and CD-Ex groups. S31, S32, S34, S36, S37, S38, S39, S42, S43, and S44 were samples in the CD-Sed group. S46, S49, S50, S51, S52, S55, S56, S57, S59, and S60 were samples in the CD-Ex group. (**B**) LEfSe analyses of the CD-Sed and CD-Ex groups. (**C**) Box plot of the top 10 different abundant genera between the CD-Sed and CD-Ex groups. (**D**) The KEGG pathway enrichment analysis based on the 16S rRNA sequencing results. (**E**) Random forest analyses of different abundant genera, top 30 genera are shown in the plot. The red-labeled text is the genus or the pathway valuable to our findings.

Gut microbiota, a critical factor in the maintenance of GI homeostasis, was also analyzed by 16S rRNA sequencing [8]. Alpha diversity, including Chao1, as well as the Simpson and Shannon indices, was calculated for each sample in the CD-Ex and CD-Sed groups (Figure 6A). Rarefaction curves reached a plateau after more than 20,000 sequences per sample, confirming sufficient sequencing depth and high data quality for subsequent analysis. No significant difference in alpha diversity was observed between the CD-Ex and CD-Sed groups. PCoA was further performed to assess beta diversity, revealing a significant difference in microbial community structure between the CD-Ex and CD-Sed groups (Figure 6A). Furthermore, LEfSe analysis identified the genus *Prevotella* as having a relatively high LDA score (Figure 6B). Among the top 10 differentially abundant genus taxa, the genus *Prevotella* exhibited the highest significance, and its random forest score was nearly two-fold or higher than that of any other genus (Figure 6C,E). Function prediction was conducted using the Kyoto Encyclopedia of Genes and Genomes (KEGG) based on the 16S rRNA sequencing results (Figure 6D). Primary bile acid biosynthesis was identified as one of the significantly downregulated pathways in exercised CRD mice.

### 3.5. Primary Bile Acid Biosynthesis Was Reduced in Exercised CRD Mice

**Figure 7:**
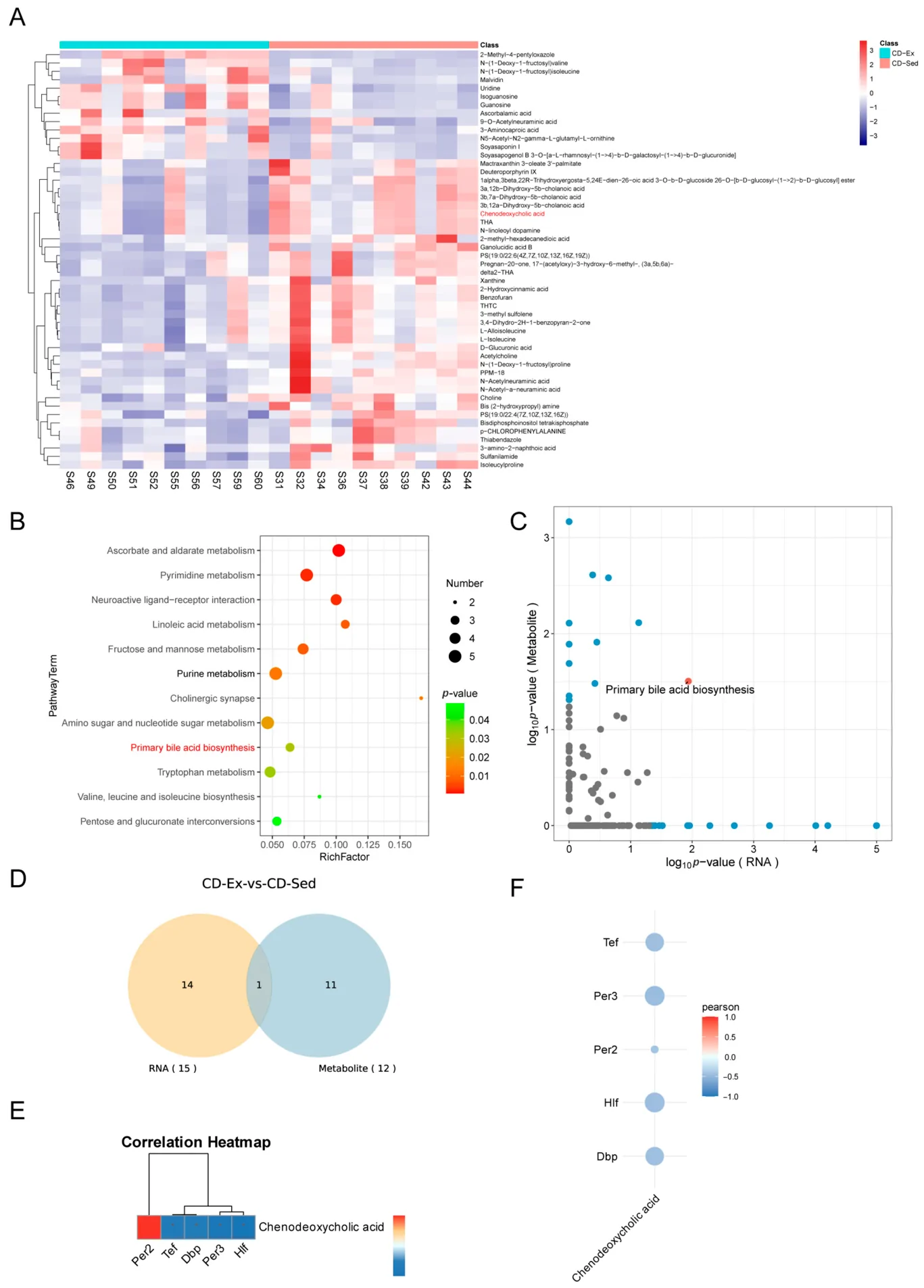
Fecal metabolome of mice in the CD-Sed and CD-Ex groups and its integrated analysis combined with the intestinal transcriptome. (**A**) Heatmap of the top 50 different metabolites. (**B**) KEGG pathway enrichment analysis of the fecal metabolome. (**C**) Integrated analysis combining the intestinal transcriptome and fecal metabolome, while the gray, blue, and red dots represent the pathways with no significance, significantly altered in either the transcriptome or metabolome, and significantly altered in both, respectively. Results of the above integrated analyses are also shown in a Venn plot (**D**). Correlation analysis between the CDCA levels and clock genes; results are shown in a cluster heatmap (**E**) and a dot plot (**F**). The red-labeled text is the metabolite or the pathway valuable to our findings.

To further explore the beneficial effects of physical exercise, the fecal metabolome of mice in the CD-Ex and CD-Sed groups was analyzed. A significant reduction in chenodeoxycholic acid (CDCA), a primary bile acid, was observed in CD-Ex mice (Figure 7A). Moreover, a significantly lower enrichment factor for primary bile acid biosynthesis was revealed by KEGG pathway enrichment analysis (Figure 7B). An integrated analysis was then performed by combining the KEGG enrichment results from the fecal metabolome and the intestinal transcriptome. As a result, primary bile acid biosynthesis was the only pathway significantly altered in both the metabolome and transcriptome (Figure 7C,D). Furthermore, a negative correlation was detected between CDCA levels and the expression of genes involved in the rhythmic process, including Tef, Hlf, Dbp, Per2, and Per3 (Figure 7E,F).

### 3.6. Reduced CDCA Biosynthesis Is Associated with Alleviated Intestinal Inflammation While Upregulated Clock Gene Expression and Abundance of Genus Prevotella Have Potential Contributions

**Figure 8:**
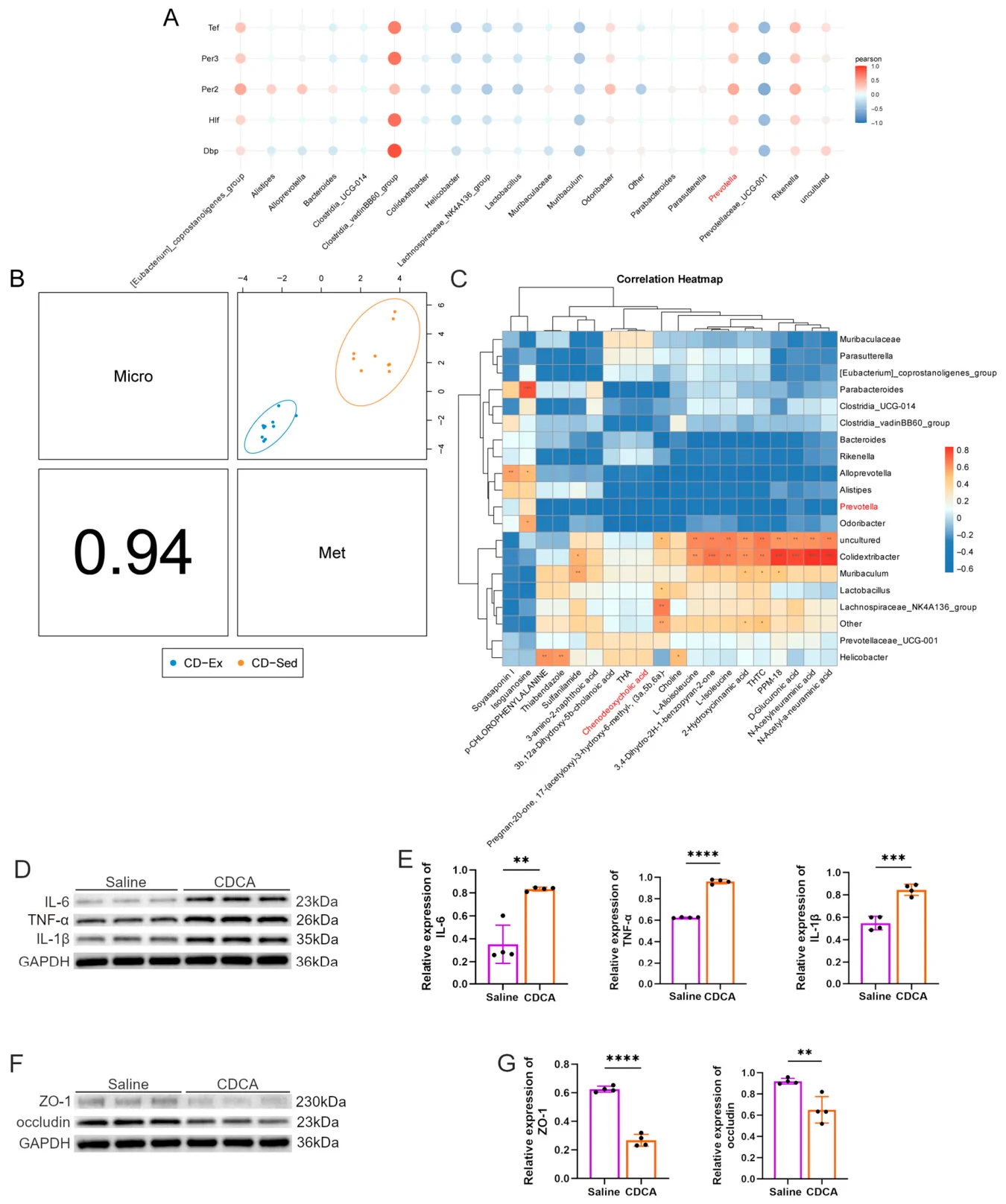
Multi-omics integration analysis combining the microbiome, transcriptome, and metabolome. (**A**) Dot plot of integration analysis between the top 20 differentially abundant genera and the expression of clock genes. (**B**) Clustering analysis of the microbiome and metabolome with a relevant correlation coefficient. The corresponding correlation heatmap is shown in (**C**). Western blotting of inflammatory factors (**D**) and intestinal barrier proteins (**F**) in the causal experiment on the effects of CDCA (*n* = 4); corresponding quantitative analysis are shown in (**E**,**G**). The red-labeled text is the metabolite or the genus valuable to our findings. * *p* < 0.05; ** *p* < 0.01; *** *p* < 0.001, and **** *p* < 0.0001.

A multi-omics integration analysis was conducted to assess correlations among the intestinal transcriptome, gut microbiota, and fecal metabolome. The results indicated that the abundance of the genus *Prevotella* was associated with the expression of clock genes, including Per2, Per3, Dbp, Tef, and Hlf (Figure 8A). Clustering analysis based on the results of the Kruskal–Wallis test from 16S rRNA sequencing and fecal metabolomics revealed no overlap, suggesting a significant difference between the CD-Ex and CD-Sed groups (Figure 8B). Furthermore, in the correlation analysis of the top 20 differentially abundant genera and fecal metabolites, a significant negative correlation was observed between the abundance of the genera *Odoribacter*, *Parabacteroides*, and *Prevotella* and CDCA levels (Figure 8C). In the causal study investigating the effect of CDCA on CRD-related intestinal inflammation, the expression of inflammatory factors, including IL-1β, IL-6, and TNF-α, was increased in the CDCA group compared with the Saline group (Figure 8D,E and Figure S29). Decreased expression of ZO-1 and occludin was also observed in exercised CRD mice administered CDCA (Figure 8F,G and Figure S30).

## 4. Discussion

Prolonged sedentary time, another characteristic of the westernized lifestyle, was associated with an increased risk of intestinal inflammation-related disease, as observed in the MR analysis. However, the marked horizontal pleiotropic effects observed for the sedentary time dataset (ukb-b-5192) suggested that other factors related to a sedentary lifestyle, such as ultra-processed food intake and alcohol consumption, may also contribute to the aggravation of intestinal inflammation [38,39]. The beneficial effects of physical exercise in ameliorating intestinal inflammation were elicited at low to mild intensities, which is consistent with findings from rodent model studies [40]. Further epidemiological investigations are warranted, given the heterogeneity observed in the mild physical activity dataset (ukb-b-7337) included in our study.

The results of the median analysis indicated that the protective effect was mediated by gut microbiota. Intestinal inflammation was exacerbated by the direct effect of moderate or vigorous intensity physical exercise, which may be attributable to the increased risk of infection and impaired intestinal barrier function [41]. Nevertheless, physical activity-induced modulation of the gut microbiota conferred substantial protection, buffering. or offsetting the underlying damage caused by high-intensity exercise. Both protective (ebi-a-GCST006098) and risk-associated (ukb-b-4710) effects were observed for moderate and vigorous physical activity, consistent with previous studies on the impact of physical exercise, particularly at moderate or higher intensities [19,41]. This paradoxical observation may arise because the overall effect of physical activity depends on the balance between the risk induced by physical exercise and the protection conferred by gut microbiota. Although no mild-intensity physical activity exposure was induced in the median analysis, the increased abundance of beneficial genera reported in animal models of voluntary exercise suggests that the protective effects of mild-intensity physical activity may also be mediated by gut microbiota [19]. The genus *Lactococcus* identified in the MR analysis may act as a protective factor against intestinal inflammation, owing to its beneficial effects on gut microbiota restoration and intestinal barrier integrity [42,43]. The MR results in our study are somewhat limited by the substantial heterogeneity or pleiotropic effects observed in most of the selected physical activity datasets. Therefore, other gut microbiota associated with physical activity may also have potential benefits in ameliorating intestinal inflammation, such as the genera *Lactobacillus*, *Bifidobacterium*, and *Prevotella* [42].

The efficacy of low-intensity physical activity in alleviating intestinal inflammation was evaluated using a CRD mouse model. Mice in the CD-Sed group exhibited marked intestinal injury, characterized by shortened villi, a thickened muscularis, and elevated levels of pro-inflammatory factors compared with healthy controls [44,45]. The protective effect of physical activity was demonstrated by attenuated intestinal injury and reduced levels of inflammatory factors in exercised CRD mice. In addition, the deposition of intestinal fibrosis, a complication of chronic intestinal inflammation, was reduced in exercised CRD mice, suggesting that physical activity alleviates the adverse consequences of chronic intestinal inflammation [46]. Furthermore, the disrupted expression of apical junctional complex proteins, including occludin and ZO-1, observed in sedentary CRD mice, was reversed by low-intensity physical exercise, indicating a beneficial role of physical activity in maintaining intestinal barrier integrity under CRD conditions [39,47]. Based on the results of the experiments from a CRD mouse model, low-intensity physical exercise exerted neither beneficial nor detrimental effects on the GI tract under basal conditions; however, it ameliorated CRD-induced intestinal inflammation and intestinal barrier impairment.

Based on the results of the MR analysis and the investigation on a CRD mouse model, sustained adequate physical activity may alleviate intestinal inflammation and its adverse effects on barrier integrity and intestinal homeostasis, with gut microbiota playing an important role. Mild and voluntary physical activity appears to have optimal efficacy in the treatment of CRD-related intestinal inflammation. The robustness of the conclusions drawn from the MR analysis should be corroborated by further studies, given some limitations of the MR analysis. First, the populations in the GWAS databases used were primarily of European ancestry, limiting the generalizability of the findings to other ethnic groups. Second, the SNP selection thresholds in the MR analysis were somewhat loose (*p* < 1 × 10^−5^), which may influence the robustness of the results. Although eight physical activity datasets were included in our analysis, only one was optimal for mediation analysis. More high-quality datasets should be employed to assess the protective effects of gut microbiota modulated by physical activity.

The intestinal tract is one of the targets of myokines secreted by skeletal muscle and is modulated by exercise-induced neurologic and vascular changes [20,48]. Here, transcriptomic analysis identified a total of 298 DEGs in exercised CRD mice, suggesting that biological processes in the GI tract were altered by physical exercise. The upregulation of brush borders (GO:0005903) in the cellular component and heat shock protein expression indicated enhanced proliferation of intestinal epithelia, suggesting that low-intensity physical activity may promote intestinal barrier integrity [49]. Nevertheless, the most notable finding in exercised CRD mice was the enhanced clock gene expression (rhythmic process, GO:0048511; circadian regulation of gene expression, GO:0032922; circadian rhythm, GO:0007623) in the GI tract. Alleviated intestinal inflammation has been reported in mice with enhanced circadian clock function induced by time-restricted feeding [6]. Thus, intestinal inflammation may be ameliorated by other pathways that enhance clock gene expression, including long-term mild-intensity physical exercise. Moreover, commensal microbiota modulated by the immune system in the GI tract under enhanced clock gene expression exert various salutary effects on intestinal homeostasis, including increased short-chain fatty acid (SCFA) production, promoted nutrient metabolism, and beneficial interactions with intestinal epithelia [1,20,50]. Positive feedback may also occur because gut microbiota could program circadian rhythms in the GI tract [30]. Although an enhanced circadian rhythm has been observed in various organs in previous studies, no myokine interacting with the circadian system has been reported in current investigations except in the brain [51]. Modulation of the GI tract by physical exercise might be mediated by sympathetic activation, splanchnic circulation during long-term physical activity, or muscle-derived exosomes that share restored circadian rhythm from myocytes [20,52]. Together, these findings suggest that physical activity could upregulate clock gene expression in the GI tract, which, in turn, alters the gut microbiota through the GI immune system, potentially contributing to the amelioration of intestinal inflammation.

The MR results suggested that gut microbiota may mediate the beneficial effects of physical exercise in ameliorating intestinal inflammation; therefore, the composition of the gut microbiota was further analyzed. Only the genera *Prevotella* and *Alloprevotella* were consistently identified by all three analytical approaches, such as LEFSe, relative abundance analysis, and random forest analysis. Collectively, the genus *Prevotella* was considered the pivotal genus contributing to the differentiation of the gut microbiota community between the CD-Ex and CD-Sed groups. An increased abundance of the genus *Prevotella* has also been reported in several previous studies investigating the relationship between physical activity and human gut microbiota [20,53]. The genus *Prevotella* is a common member of the gut microbiota in humans and other mammals, comprising more than 50 species, and has been associated with multiple effects, exerting both beneficial and detrimental ones [54]. The beneficial effect of the genus *Prevotella* may be mediated by probiotic species and by their complex metabolic activities and interactions with host mucosal immunity, such as SCFA production and glucose homeostasis [54]. In addition, functional prediction revealed a decrease in biosynthesis of primary bile acids, which is consistent with previous observations and has been associated with exacerbated inflammation [55].

The genus *Lactococcus* was identified as a potential mediator in the MR analysis, whereas the results of 16S rRNA sequencing and analysis suggested that the genus *Prevotella* may serve as an important mediator. The genus *Prevotella* tends to dominate the gut microbiome in populations following a pre-industrial lifestyle [54]. The populations included in the MR datasets are primarily of European ancestry who have been living modern lifestyles and consuming Western diets for more than a century. Therefore, the MR results may be influenced by the relatively low prevalence and abundance of the genus *Prevotella* in European populations. Moreover, the gut microbiota datasets used in the MR analysis are based on 16S rRNA gene sequencing profiles in which both probiotic and pathogenic species of *Prevotella* were combined, leading to the detection of both protective and risk-associated effects for the abundance of the genus *Prevotella* in the MR analysis (Table S5). On the other hand, certain species of *Lactococcus* are common fermenting bacteria used in food production, particularly in dairy products such as cheese [56]. The genus *Lactococcus* may therefore have a relatively rich abundance in European populations due to a high consumption of fermented dairy products, which could explain why its protective effect was readily detected and statistically significant in the MR analysis. Furthermore, the genus *Lactococcus* may tend to interact or cross-feed with other microorganisms in the intestinal tract [56]. Its protective effect may be mediated though the promotion of other microorganisms, possibly including some beneficial species of the genus *Prevotella*. In summary, the community structure of the gut microbiota can be modulated by physical exercise, and the genera *Prevotella* and *Lactococcus* appear to contribute beneficial effects in ameliorating CRD-related intestinal inflammation. However, the exact role of gut microbiota should be further investigated to determine whether it functions as an important mediator or as a beneficial outcome of the effects of physical exercise.

CDCA is a principal component of primary bile acids in humans and is known for its role in the digestion and absorption of lipids and carbohydrates [57]. Primary bile acids can be metabolized by gut microbiota into secondary bile acids, which play important roles in the regulation of metabolism, innate immunity, and intestinal homeostasis [57,58]. Administration of CDCA or a medicine that increases production of primary bile acids may alleviate intestinal inflammation through the beneficial regulation of secondary bile acids [59,60]. Nevertheless, elevated levels of CDCA were observed in sedentary CRD mice and were reversed by physical exercise. This paradoxical finding may be attributable to disrupted intestinal homeostasis during chronic gastrointestinal disorders [61]. Levels of primary bile acids in intestinal contents may be elevated as a result of impaired secondary bile acid biosynthesis and enterohepatic circulation [58,61]. Furthermore, CDCA is characterized as a detergent due to its amphiphilic molecular structure, and it can cause mucosal damage by inducing mucus denudation from the surface of the colonic mucosa, thereby aggravating intestinal inflammation [62]. Increased mucosal permeability and decreased occluding expression mediated by CDCA have also been reported in previous studies [62]. In addition, CDCA acts as an agonist of the farnesoid X receptor and suppresses Wnt signaling in intestinal stem cells, thereby promoting intestinal damage [63]. The elevated inflammatory factor levels and reduced expression of apical junctional complex proteins observed in the CDCA group demonstrate that increased CDCA levels could aggravate CRD-related intestinal inflammation. CDCA appears to exert more detrimental effects during CRD, which induces dysregulation of the gastrointestinal system; therefore, reduced primary bile acid biosynthesis could contribute to the attenuation of CRD-related intestinal inflammation.

The multi-omics integration analysis corroborated associations among the circadian rhythm, gut microbiota, and fecal metabolites, thereby revealing a potential pathway underlying the beneficial effect of physical exercise. First, clock gene expression in the GI tract was enhanced by long-term low-intensity physical exercise. Enhanced clock gene expression in the GI tract may promote the proliferation of intestinal epithelia and regeneration of the intestinal barrier, thereby contributing to the alleviation of intestinal inflammation-induced damage. Second, the community structure of the gut microbiota was modulated by interactions between the GI tract, with enhanced clock gene expression and physical exercise-induced stimuli. The abundance of the genus *Prevotella* was critical to the structural differentiation of the gut microbiota between exercise-trained and sedentary lifestyles, and it was positively associated with various genes involved in the circadian process. Third, the association between alterations in gut microbiota structure and variations in the fecal metabolome was illustrated, with CDCA levels in the intestinal contents being primarily associated with the genus *Prevotella* due to its dominance in the exercise-modulated differentiation of the gut microbiota. The association between reduced CDCA levels and alleviated intestinal inflammation was further corroborated by the results of CDCA administration in CD-Ex mice.

The present investigation of the anti-inflammatory efficacy of physical exercise has several limitations that warrant further exploration. First, an unadjusted *p*-value was used in the intestinal transcriptome analysis to enhance sensitivity for identifying potential pathways underlying the benefits of physical exercise. Most associations identified in the multi-omics integration analysis were limited by a lack of causal evidence and therefore remain hypothesis-generating. Some stronger causal studies are required to validate the association among physical exercise, circadian rhythm, gut microbiota, and other potential corresponding metabolites. The effect of CDCA was explored in a preliminary experiment with four mice per group; this finding should be further evaluated in a larger-scale study. Furthermore, the robustness of the multi-omics integration analysis was limited by a somewhat small number of mice in each group. The genus *Lactococcus* was identified as a potential mediator by the MR analysis based on human databases, whereas the genus *Prevotella* was suggested as a mediator by the gut microbiota analysis in exercised CRD mice. Therefore, the disparity between the MR and microbiome findings should be clarified through investigations using human samples, such as cohort studies. Aside from that, specific species of *Prevotella* could not be determined due to accuracy limitations of 16S rRNA sequencing. If the mediator role of the genus *Prevotella* is confirmed, then the detailed interactions between this genus and the host GI tract should be further explored using advanced analysis methods for the transcriptome of the GI tract and genome of the genus *Prevotella*.

## 5. Conclusions

Our study suggests that CRD-related intestinal inflammation could be ameliorated by physical exercise, and this efficacy is associated with reduced biosynthesis of CDCA. The beneficial effect of physical exercise may also be linked to enhanced clock gene expression in the GI tract, and to alterations in gut microbiota, in which the genera *Prevotella* and *Lactococcus* may play important roles. A novel study direction about the treatment of gastrointestinal disorders caused by CRD-related intestinal inflammation may be illuminated by the anti-inflammatory effect of physical exercise and its association with CDCA identified in this study. The importance of keeping adequate physical activity throughout life in modern society is underlined, with a viewpoint that exercise is medicine.

## Figures and Tables

**Table 1:** MR results of causality between physical activity and intestinal inflammation (method: IVW).

id.exposure	nsnp	β	se	*p*-Value	OR (95% CI)	Steiger *p*-Value	Q *p*-Value	P *p*-Value
ebi-a-GCST006097	24	6.14 × 10^−2^	2.17 × 10^−2^	4.65 × 10^−3^	1.063 (1.019-1.110)	1.88 × 10^−53^	3.63 × 10^−1^	1.07 × 10^−2^
ebi-a-GCST006098	93	−4.00 × 10^−2^	1.79 × 10^−2^	2.52 × 10^−2^	0.961 (0.928-0.995)	8.87 × 10^−166^	6.90 × 10^−3^	3.20 × 10^−1^
ebi-a-GCST006099	78	−8.32 × 10^−4^	7.47 × 10^−4^	2.66 × 10^−1^	0.999 (0.998-1.001)	1.34 × 10^−246^	9.92 × 10^−2^	8.35 × 10^−1^
ebi-a-GCST90061421	61	−1.19 × 10^−3^	5.13 × 10^−3^	8.17 × 10^−1^	0.999 (0.989-1.009)	4.24 × 10^−194^	1.34 × 10^−1^	6.43 × 10^−1^
ukb-b-151	83	−1.10 × 10^−2^	5.88 × 10^−3^	6.21 × 10^−2^	0.989 (0.978-1.000)	1.47 × 10^−113^	1.26 × 10^−1^	7.13 × 10^−1^
ukb-b-4710	22	3.22 × 10^−2^	9.33 × 10^−3^	5.63 × 10^−4^	1.033 (1.014-1.052)	3.72 × 10^−49^	7.29 × 10^−1^	1.96 × 10^−1^
ukb-b-5192	445	3.39 × 10^−2^	7.27 × 10^−3^	3.16 × 10^−6^	1.034 (1.020-1.049)	<0.001	5.97 × 10^−5^	1.22 × 10^−2^
ukb-b-7337	148	−5.83 × 10^−2^	2.15 × 10^−3^	6.74 × 10^−3^	0.943 (0.904-0.984)	1.72 × 10^−171^	8.95 × 10^−3^	7.20 × 10^−1^

Note: nsnp: number of SNPs, β: effect size; se: standard error; CI: confidence interval.

**Table 2:** MR results of causality between gut microbiota (*p*-Val < 5 × 10^−2^) and intestinal inflammation (method: IVW).

id.exposure	nsnp	β	se	*p*-Value	OR (95% CI)	Steiger *p*-Value	Q *p*-Value	P *p*-Value
ebi-a-GCST90017116	12	1.27 × 10^−1^	6.36 × 10^−3^	4.66 × 10^−2^	1.013 (1.000-1.025)	5.36 × 10^−58^	5.24 × 10^−1^	9.01 × 10^−1^
ebi-a-GCST90016990	11	−1.02 × 10^−1^	4.36 × 10^−3^	1.95 × 10^−2^	0.990 (0.981-0.998)	9.46 × 10^−46^	6.95 × 10^−1^	2.37 × 10^−1^
ebi-a-GCST90017052	9	1.38 × 10^−1^	6.52 × 10^−3^	3.37 × 10^−2^	1.014 (1.001-1.027)	1.09 × 10^−53^	7.89 × 10^−1^	6.29 × 10^−1^
ebi-a-GCST90016924	7	−1.37 × 10^−2^	5.82 × 10^−3^	1.87 × 10^−2^	0.986 (0.975-0.998)	6.10 × 10^−34^	5.03 × 10^−1^	6.32 × 10^−1^
ebi-a-GCST90017031	6	−1.11 × 10^−1^	5.13 × 10^−3^	3.09 × 10^−2^	0.989 (0.979-0.999)	1.32 × 10^−37^	4.12 × 10^−1^	8.04 × 10^−1^
ebi-a-GCST90017005	15	−1.23 × 10^−1^	5.52 × 10^−3^	2.60 × 10^−2^	0.988 (0.977-0.999)	3.92 × 10^−74^	6.88 × 10^−1^	9.16 × 10^−1^
ebi-a-GCST90016912	12	1.30 × 10^−1^	6.46 × 10^−3^	4.49 × 10^−2^	1.013 (1.000-1.026)	7.78 × 10^−66^	8.18 × 10^−1^	4.44 × 10^−1^
ebi-a-GCST90017086	14	−8.99 × 10^−2^	4.31 × 10^−3^	3.70 × 10^−2^	0.991 (0.983-0.999)	2.48 × 10^−64^	6.05 × 10^−1^	6.56 × 10^−1^

**Table 3:** MR results of causality between physical activity (ukb-b-4710) and gut microbiota (ebi-a-GCST90017031) (method: IVW).

id.exposure	nsnp	β	se	*p*-Value	OR (95% CI)	Steiger *p*-Value	Q *p*-Value	P *p*-Value
ukb-b-4710	99	1.96 × 10^−1^	9.10 × 10^−2^	3.10 × 10^−2^	1.217 (1.018-1.455)	5.67 × 10^−3^	8.55 × 10^−1^	6.17 × 10^−1^

## Data Availability

The original contributions presented in this study are included in the article/Supplementary Material. Further inquiries can be directed to the corresponding authors.

## Supplementary Materials

The following supporting information can be downloaded at: https://www.mdpi.com/article/10.3390/cells15171537/s1, Table S1: Characteristics of the 954 SNPs from datasets of physical activity associated with intestinal inflammation; Table S2: Characteristics of the 86 SNPs from datasets of gut microbiota associated with intestinal inflammation; Table S3: Characteristics of the 99 SNPs from physical activity (ukb-b-4710) associated with abundance of genus *Lactococcus* (ebi-a-GCST90017031); Table S4: Statistically significant DEGs with adjusted *p*-values using Benjamini–Hochberg method; Table S5: Statistics of MR analysis on causality between genus *Prevotella* and intestinal inflammation; Figures S1–S8: Scatter plots of MR analysis on causality between datasets of physical activity and intestinal inflammation while SNP effects were shown on outcome (Y-axis, IBD || id: GCST90014444) and exposure (X-axis) with 95% confidence intervals; S1: Moderate to vigorous physical activity levels || id:ebi-a-GCST006097; S2: Vigorous physical activity || id:ebi-a-GCST006098; S3: Accelerometer-based physical activity measurement (average accelera-tion) || id:ebi-a-GCST006099; S4: Light-intensity physical activity duration || id:ebi-a-GCST90061421; S5: Number of days/week of vigorous physical activity 10+ min || id:ukb-b-151; S6: Number of days/week of moderate physical activity 10+ min || id:ukb-b-4710; S7: Time spent watching television (TV) || id:ukb-b-5192; S8: Types of physical activity in last 4 weeks: Walking for pleasure (not as a means of transport) || id:ukb-b-7337; Figure S9: Funnel plot for physical activity (ukb-b-4710) on intestinal inflammation; Figure S10: Forest plot on MR effect size for physical activity (ukb-b-4710) on intestinal inflammation; Figure S11: Leave-one-out forest plot for physical activity (ukb-b-4710) on intestinal inflammation; Figures S12–S19: Scatter plots on causality between datasets of gut microbiota and intestinal inflammation; S12: Gut microbiota abundance (phylum *Proteobacteria* id.2375) || id:ebi-a-GCST90017116; S13: Gut microbiota abundance (genus *Eggerthella* id.819) || id:ebi-a-GCST90016990; S14: Gut microbiota abundance (genus *Ruminococcaceae* NK4A214 group id.11358) || id:ebi-a-GCST90017052; S15: Gut microbiota abundance (family *Acidaminococcaceae* id.2166) || id:ebi-a-GCST90016924; S16: Gut microbiota abundance (genus *Lactococcus* id.1851) || id:ebi-a-GCST90017031; S17: Gut microbiota abundance (genus *Eubacterium ventriosum* group id.11341) || id:ebi-a-GCST90017005; S18: Gut microbiota abundance (class *Betaproteobacteria* id.2867) || id:ebi-a-GCST90016912; S19: Gut microbiota abundance (unknown genus id.826) || id:ebi-a-GCST90017086; Figure S20: Scatter plot on physical activity (ukb-b-4710) on abundance of genus *Lactococcus* (ebi-a-GCST90017031); Figure S21: Funnel plot on abundance of genus *Lactococcus* (ebi-a-GCST90017031) on intestinal inflammation; Figure S22: Forest plot on MR effect size for abundance of genus *Lactococcus* (ebi-a-GCST90017031) on intestinal inflammation; Figure S23: Leave-one-out forest plot on abundance of genus *Lactococcus* (ebi-a-GCST90017031) on intestinal inflammation; Figure S24: Funnel plot on physical activity (ukb-b-4710) on abundance of genus *Lactococcus* (ebi-a-GCST90017031); Figure S25: Forest plot on MR effect size for physical activity (ukb-b-4710) on abundance of genus *Lactococcus* (ebi-a-GCST90017031); Figure S26: Leave-one-out forest plot on physical activity (ukb-b-4710) on abundance of genus *Lactococcus* (ebi-a-GCST90017031); Figure S27: Uncropped image for Western blotting analysis on IL-6, TNF-α, and IL-1β in groups LD-Sed, LD-Ex, CD-Sed, and CD-Ex; Figure S28: Uncropped image for Western blotting analysis on ZO-1 and occludin in groups LD-Sed, LD-Ex, CD-Sed, and CD-Ex; Figure S29: Uncropped image for Western blotting analysis on IL-6, TNF-α, and IL-1β in groups Saline and CDCA; Figure S30: Uncropped image for Western blotting analysis on ZO-1 and occludin in groups Saline and CDCA.

## Abbreviations

The following abbreviations are used in this manuscript:

CRD	Circadian rhythm disruption
GI tract	Gastrointestinal tract
MR	Mendelian randomization
SNPs	Single nucleotide polymorphisms
OR	Odds radio
PCoA	Principal coordinates analysis
LEFSe	Linear discriminant analysis effect size
CDCA	Chenodeoxycholic acid

## Appendix A

**Table A1:** Datasets of physical activities and intestinal inflammation involved in MR study.

Exposure (Trait/Dataset Type)	GWAS ID	Consortium/Author	Population	Sample Size
Sedentary behavior: time spent watching television (TV)	ukb-b-5192	MRC-IEU	European	437,887
Low-intensity exercise: types of physical activity in last 4 wk: walking for pleasure	ukb-b-7337	MRC-IEU	European	460,376
Moderate-intensity exercise: number of days/week of moderate physical activity 10+ min	ukb-b-4710	MRC-IEU	European	440,266
High-intensity exercise: number of days/week of vigorous physical activity 10+ min	ukb-b-151	MRC-IEU	European	440,512
Average physical activity	ebi-a-GCST006099	Klimentidis YC	European	91,084
Light physical activity	ebi-a-GCST90061421	Qi G	European	88,411
Moderate to vigorous physical activity	ebi-a-GCST006097	Klimentidis YC	European	377,234
Vigorous physical activity	ebi-a-GCST006098	Klimentidis YC	European	98,060 cases and 162,995 controls
Inflammatory bowel disease	GCST90014444	Glanville KP	European	5105 cases, 324,074 controls

**Table A2:** Primers used in real-time qPCR.

Gene	Forward	Reverse
Gapdh	CCTCCTCCAATTCAACCCT	ATCCGTTCACACCGACCT
ZO-1	ACCATGCCTAAAGCTGTCC	GGAACTCAACACACCACCA
Occludin	CAATGGCCTACTCCTCCA	TGCCTGAAGTCATCCACA
IL-1β	TGAGGACATGAGCACCTTC	GGGAACGTCACACACCA
IL-6	GCAAGAGACTTCCATCCAGT	GGTTGTCACCAGCATCAGT
TNF-α	GGCAGGTTCTGTCCCTTT	TGCTTTCTGTGCTCATGGT

**Table A3:** Antibodies used in this study.

Antibody	Art.No.	Dilution
Anti-GAPDH Rabbit Recombinant Monoclonal Antibody	GB15004	WB: 1:5000
Anti-ZO1 tight junction protein Rabbit Recombinant Monoclonal Antibody	GB15195	WB: 1:2000; IF: 1:300
Anti-Occludin Mouse Recombinant Monoclonal Antibody	GB15149	WB: 1:500
Anti-IL-1 beta Rabbit Recombinant Monoclonal Antibody	GB15115	WB: 1:800
Anti-IL-6 Rabbit Polyclonal Antibody	GB11117	WB: 1:1000
Anti-TNF-alpha Rabbit Recombinant Monoclonal Antibody	GB153968	WB: 1:2000
Anti-iNOS Rabbit Polyclonal Antibody	GB11119	IF: 1:500
HRP-conjugated Goat Anti-Rabbit IgG (H+L)	GB23303	WB: 1:5000
Cy3-conjugated Goat Anti-Rabbit IgG (H+L)	GB21303	IF: 1:300

All antibodies used in this study were purchased from Servicebio Co., Ltd. (Wuhan, Hubei, China). WB: Western blotting. IF: immunofluorescence stain.
